# A New Viscous Budesonide Formulation for the Treatment of Eosinophilic Esophagitis in Children: A Preliminary Experience and Review of the Literature

**DOI:** 10.3390/jcm11226730

**Published:** 2022-11-14

**Authors:** Joanna Warzecha, Marcin Dziekiewicz, Alicja Bieńkowska-Tokarczyk, Maciej Małecki, Aleksandra Banaszkiewicz

**Affiliations:** 1 Department of Pediatric Gastroenterology and Nutrition, Medical University of Warsaw, 02-097 Warsaw, Poland; 2 Department of Applied Pharmacy, Faculty of Pharmacy, Medical University of Warsaw, 02-097 Warsaw, Poland

**Keywords:** eosinophilic esophagitis, oral viscous budesonide, topical steroids, formulation, treatment, viscous slurry

## Abstract

Eosinophilic esophagitis (EoE) is a chronic disease, characterized clinically by esophageal disfunction. Topical corticosteroids (tCS), predominantly fluticasone and budesonide, are considered the effective first line treatment, as well as an option of maintenance therapy in EoE. The way that tCS are administered significantly affects their effectiveness. There is still no ready-to-use steroid drug to be applied topically to the esophagus in children—a few experimental viscous slurries (mainly of budesonide) have been shown in trials to be more effective than steroids administered via metered dose inhalers (MDIs) and swallowed. The best examined steroid solvent of all is sucralose, a high-intensity artificial sweetener. Although it has been shown in a critical review that it is non-toxic and safe for all consumers, there are still some concerns among patients about its potential adverse effect on humans. Due to that fact, we developed a new viscous formulation and evaluated its effectiveness in the treatment of children with EoE. In an open, prospective, single-center study, we administered our new formulation of viscous budesonide twice daily for 8 weeks in patients with an active EoE. After treatment, we performed a control gastroscopy with the collection and evaluation of histopathological samples. We have proven our formulation effectiveness at 64%, as far as histological remission is concerned. We have also shown a reduction in the mean endoscopic reference score (EREFS) from 3.1 points at the beginning of the study to 1.6 points at the end of the study. Bearing in mind how important the acceptance of the solvent is for long-time compliance, especially among children, we also decided to assess the taste of the formulation. Therefore, we asked 46 adults and 10 children to swallow a sample of the solvent and fill in a short anonymous questionnaire about its taste, smell, consistency and easiness of swallowing. General acceptance for the proprietary solvent was high, reaching 7.5/10 among adults and 6.5/10 in children. To be able to compare the results of our preliminary experience, we reviewed the studies which evaluated substances that have been used so far as steroid solvents for the treatment of EoE. The overall effectiveness of the oral viscous budesonide (OVB) ranged from 65% to 90%, which is consistent with the results obtained in our study. Unfortunately, the high heterogeneity of the studies did not allow us to draw reliable conclusions.

## 1. Introduction

Eosinophilic esophagitis (EoE) is a chronic, multifactorial, immune-mediated disease characterized clinically by esophageal dysfunction resulting from mucosal inflammation. Histologically, it manifests by eosinophilic infiltration with a count of ≥15 eosinophils per high-power field (HPF) [1]. Chronic inflammation leads to progressive esophageal fibrosis, strictures, and narrowing. The first cases of EoE were reported in the 1970s, and since then, its prevalence and incidence have continually increased, making EoE the leading cause of esophageal dysfunction and food impaction among young adults and children [2].

Clinical symptoms of EoE vary with age. Adults and adolescents usually present with dysphagia, heartburn, chest pain, or food impaction, whereas infants and children present more commonly with symptoms of feeding problems, failure to thrive, or vomiting. Some patients may also manifest respiratory symptoms, such as cough or sleep disorders [3]. Treatment of EoE includes three equal interventions: elimination diet or one of the two possible medical therapies—proton pump inhibitors or topically acting corticosteroids. In case of complications, such as strictures, endoscopic intervention may be required [4].

Topical corticosteroids (tCS), predominantly fluticasone and budesonide, are considered the most effective first-line treatment, as well as an option for maintenance therapy in EoE [5,6]. These drugs induce histological remission, lead to the resolution of clinical symptoms, and prevent fibrosis. The way that topical corticosteroids are administered significantly affects their effectiveness [7]. There is no steroid drug to be applied topically to the esophagus in children. For adults with EoE, budesonide orodispersible tablets (i.e., Jorveza*^®^*) were recently designed. However, although Jorveza^®^ is effective, it is not widely available. Moreover, a liquid budesonide oral suspension is currently still in a phase III trial in the United States (Takeda) [8].

In children, due to the lack of other possibilities, swallowed metered-dose inhalers (MDI) steroids are used. Another possibility for topical budesonide to be used in the esophagus is by combining it with another substance. Until now, only a few experimental viscous slurries (mainly of budesonide) have been shown in trials to have greater mucosal contact, and hence, to be more effective than steroids administered via MDI or swallowed [5].

An ideal diluent for topical corticosteroids used in EoE should meet certain features: it should be safe and hypoallergic, ensure long mucosal contact, and have an acceptable taste. The best-examined steroid solvent of all is sucralose—a product of sucrose, a high-intensity sweetener, which is not metabolized and does not affect blood glucose levels [9]. The biggest disadvantage of sucralose as a drug solvent is that, although it is devoid of calories, it is an artificial sweetener. This is a concern of some patients or their parents because they are unsure of its impact on an organism after daily use. However, Magnuson et al. have shown in their critical review that sucralose is non-toxic, non-carcinogenic, and safe for all consumers [9].

The possibility of offering patients alternative solutions would give us a chance of involving the patient in the process of making therapeutic decisions, which increases his sense of responsibility for his treatment, and thus increases the chance of good compliance in the future. To treat our patients with EoE budesonide topically as effectively as possible, we developed a new viscous formulation. In a pilot study, we evaluated its effectiveness in the treatment of children with EoE. To be able to compare the results of our pilot study, we reviewed studies that evaluated substances used to date as a steroid solvent for the treatment of EoE.

## 2. The New Formulation

### 2.1. Methods

The new formulation was invented by our team of researchers, which included clinicians and pharmacists in the Faculty of Pharmacy of the Medical University of Warsaw, Poland as previously described [10]. We aimed to create a formulation that was maximally sticky, safe, hypoallergic (as EoE is frequently diagnosed in allergic patients), inexpensive, and easy to make in any pharmacy. Briefly, an emulsion, liquid formulation containing polysaccharides and oily excipients was obtained. Budesonide was chosen as the active substance. The stability and persistence of the developed formulation stored at room temperature and 4 °C were evaluated. Cylinder, pendulum, dose capacity, and coating tests were carried out. Its pH, viscosity, and osmolarity were assessed. The viscosity of the budesonide formulation has been shown to be about 25 times higher and the coating time was about 11 times longer than that of pure budesonide. The budesonide formulation was stable at 4 °C for up to 31 days. The development of the formulation was supported by the Innovation Incubator 2.0 (grant no. 10/2019/CTT).

We evaluated the taste of the formulation among several groups of people. We asked doctors, nurses, medical students, and patients with EoE to swallow a sample of our formulation and complete a short anonymous questionnaire about its taste, smell consistency, and easiness of swallowing. Additionally, we asked about general acceptance of the formulation and if they would agree to swallow the formulation for 8 weeks daily. All ratings were on a scale from 1 to 10 points, with 1 point being unacceptable and 10 points being excellent. The evaluation was conducted in the Department of Pediatric Gastroenterology and Nutrition, Medical University of Warsaw, Poland.

### 2.2. Results

In total, 56 people participated in the study (46 adults and 10 children). Participants rated the taste of the solution the worst, while they felt its smell and texture were more tolerable. The median score for the taste was 4/10 among both adults and children. General acceptance for the proprietary solvent was high, reaching a median score of 7.5/10 among adults and 6.5/10 among children. Notably, only three children refused to use our slurry for 8 weeks and four for 12 weeks, regardless of whether it was taken once or twice a day. The acceptance in the adult group was a bit higher and exceeded 90% when taken once a day. The detailed results concerning the acceptance of our formulation in all groups are shown in Table 1.

## 3. Effectiveness and Safety of the New Budesonide Formulation in Children with EoE: The Pilot Study

### 3.1. Methods

We conducted an open-label (without a control group), prospective, single-center study at the Department of Paediatric Gastroenterology and Nutrition, Medical University of Warsaw, Poland between 2019 and 2020. Research participants were recruited among children diagnosed with EoE based on standard, clinical, endoscopic, and histologic findings (≥15 eosinophils high power field [HPF] in any of the esophageal mucosa biopsies). Eleven participants were divided into two groups based on height: group A ≥150 cm and group B < 150 cm. The two groups received 2 mg of budesonide/6 mL formulation or 1 mg of budesonide/3 mL formulation, respectively. The budesonide formulation was administered twice daily for 8 weeks. Participants were asked to prepare the viscous slurry by themselves, just before administration, by mixing one or two ampules of 2 mL of budesonide solution for nebulization (0.5 mg/mL) with the appropriate volume of proprietary solvent, depending on the group (6 mL for group <150 cm and 10 mL for patients ≥150 cm). After swallowing the slurry, patients were asked not to eat or drink for at least 30 min. At the time of enrollment and in the 8th week of the study, patients underwent a gastroscopy. EoE severity was determined using the eosinophilic esophagitis reference score (EREFS) that assesses the following findings: edema (0–2 points), exudates (0–2 points), furrows (0–2 points), rings (0–3 points), and strictures (0–1 points) [11]. At least two mucosal biopsies from all three levels of the esophagus (upper, middle, and lower) were also obtained and examined at the Department of Pathology, Medical University of Warsaw, to assess the presence and intensity of eosinophilic infiltration of the mucosa. Histological findings were scored with values of peak intraepithelial eosinophils/hpf under light microscopy (×400). The remission of EoE was defined as an eosinophil count <15/hpf in all biopsies. Patients were also asked to complete a proprietary questionnaire on the severity of clinical symptoms. Clinical symptoms were assessed in the 8th week of the study. Cortisol levels were measured in the study participants at the beginning and the end of the treatment. The primary outcome measure was the number of patients who achieved remission after 8 weeks of treatment.

### 3.2. Results

In total, we enrolled 13 patients who met the inclusion criteria. All agreed to participate in the study, but two did not complete the trial: one did not report for a follow-up visit and the second did not take medications regularly. Finally, 11 male children aged 5 to 17 years (median age = 11 years) were included in the analysis (Table 2). Six children were newly diagnosed with EoE at the time of their enrollment in the study. In the 8th week of the study, 7/11 (64%) patients achieved histological remission. Among the four patients who did not achieve histological remission, 2/4 (50%) reduced the number of eosinophils infiltrating the mucosa, and 1/4 reported a significant reduction in clinical symptoms. The mean severity of endoscopic EoE decreased from 3.1 points at the beginning of the study to 1.6 points at the end of the study as assessed by eosinophilic esophagitis reference scores. During the study, no side effects of the preparation used were found, whether subjective, reported by participants or objective (e.g., esophageal candidiasis). Serum cortisol concentration was measured in six of the participants at the end of the treatment. In one case, it was slightly elevated but normalized in several weeks, while it was within referenced range for the other five participants.

## 4. Oral Viscous Budesonide Formulation in the Treatment of EoE: A Review

We performed a search of the MEDLINE database for studies investigating the efficacy of oral viscous budesonide (OVB) slurries in EoE. In the search, we used the following keywords: EoE, oral budesonide, viscous slurry, and topical steroids. We collected data on the type of slurry, number of patients, dose and duration of therapy, histological response, clinical improvement, and, if possible, taste assessment.

In total, we found 14 studies (733 participants) that assessed the effectiveness of various budesonide slurries in the treatment of EoE. Of them, nine were performed exclusively or partially in a child population (323 participants). In most trials (9/14), the OVB slurry was made using 5 to 10 g of sucralose mixed with 4 to 8 mL of budesonide solution intended for nebulized administration (0.5 mg/2 mL). The final volume of a single dosage of the solution was between 8 and 10 mL and the concentration was about 0.2 mg/mL. Some studies analyzed different solvents, such as xanthan gum, xylitol, honey, infant formulas, or apple sauce; for the sake of clarity, we have presented them in separate lines. The effectiveness of the OVB ranged from 65% to 90%; however, the high heterogeneity of the studies did not allow us to draw reliable conclusions. Detailed data are presented in Table S1 of Supplementary Materials.

## 5. Discussion

The effectiveness of OVB in inducing and maintaining the remission of EoE has been proven in many clinical trials. Our study found histopathological remission (<5 eos/HPF) in 64% of patients (7/11) and response to treatment in 81% (9/11). This is slightly lower than in the study by Oliva et al. [12], which, like ours, was an observation pilot trial. Microscopically confirmed remission was achieved by 88.9% of patients (32/36). This difference is probably related to the small size of the study population, which is a common feature of many trials on EoE, especially performed in more defined populations (e.g., pediatrics). The effectiveness of OVB is associated not only with the reduction in microscopic lesions but also with the severity of clinical symptoms and endoscopic lesions [13]. A recently published systematic review of the literature with meta-analysis revealed that OVB significantly increases the probability of achieving histopathological remission of EoE compared to a placebo (RR 23.82, 95% CI: 13.46–42.21, *p* < 0.001) [14] as well as a reduction in the severity of clinical symptoms (RR 1.72, 95% CI: 1.22–2.41, *p* = 0.002 and MD 2.45, 95% CI: 0.76–4.15, *p* = 0.005) [15].

The effect of OVB treatment appears to be quite fast. Straumann et al., after only 15 days of treatment with a medium dose of OVB (1.0 mg 2× daily), found statistically significant differences from a placebo in terms of both the severity of dysphagia (5.61 vs. 2.22; *p* < 0.001) and the reduction in eosinophilic infiltration (from 68.2 to 5.5 eos/HPF, *p* < 0.0001 for OVB and from 62.3 to 56.5 eos/HPF, *p* = 0.48 for placebo) [16].

The effectiveness of OVB treatment is partially dose-dependent. Most studies used a cumulative daily dose of 4 mg in older children and adults. Gupta et al. studied whether dose reduction affected the effectiveness of the treatment [17]. Daily doses of 0.5 mg, 2 mg, and 4 mg (in children over 10 years of age) were compared with a placebo. Histological remission was achieved in a significantly higher percentage of children compared to placebo in the medium- and high-dose groups but not the lowest dose. Interestingly, the reduction in the severity of clinical symptoms was similar in all subjects, including the placebo group. The small sample size in different study subgroups did not allow for the comparison of OVB doses with each other.

OVB was contrasted not only with a placebo. In an interesting study, Dohill et al. compared OVB and a placebo while using lansoprazole in both groups [5]. After 12 weeks, 86.7% of the OVB + lansoprazole group achieved histopathological remission and, interestingly, no one in the control group. However, the difference was not statistically significant (*p* = 0.30). OVB was also compared in head-to-head trials with fluticasone. Despite numerous theoretical premises favoring OVB, no statistically significant differences were found in the effectiveness of both steroids [18,19] or the frequency of relapse after treatment discontinuation [20].

OVB has also been shown to be effective in maintaining EoE remission. When such treatment was continued after successful induction of remission, the relapse rate after 36 weeks was significantly lower compared to a placebo (24.0% vs. 43.5%, *p* = 0.131) [21]. Importantly, in the cited study, the drug dose from the induction of the remission phase was continued in the maintenance phase (2.0 mg b.i.d.). In another study in the second phase of treatment, a reduced dose of OVB (0.25 mg b.i.d.) was used for 50 weeks [22]. Once again, a positive treatment effect was found when compared to a placebo (relapse rate 50% vs. 71.4%, complete remission rate 35.7% vs. 0%, *p* = 0.647). Similar conclusions were drawn from another study [23]. An observational pediatric trial was also conducted [24]. After 12 weeks of induction of remission with OVB, it was achieved in 90% of the subjects. After a dose reduction by half from baseline and continuation of treatment, remission was maintained in 85% and 45% of subjects after 24 weeks and 36 weeks, respectively.

An important aspect of the analyzed treatment is patients’ compliance. Since better taste can potentially improve compliance, especially among children, we decided to analyze the taste, smell, and consistency of our slurry. The median general acceptance of the formulation in the group of patients with EoE was 6.5/10 points. Surprisingly, better acceptance was found in the group of younger children (8/10 points). Importantly, they maintained their acceptance of the formulation for the 8 or 12 weeks of treatment. To the date of publication, only one trial had assessed the taste of viscous slurries. In the Hefner et al. study, adult volunteers assessed the tolerability of a single dose of three slurries mixed with budesonide: sucralose, honey, and xanthan gum [25]. Validated hedonics generalized labeled magnitude scale was used for the assessment. The scale ranges from −100 (strongest imaginable disliking of any kind ever experienced) to +100 (strongest imaginable liking of any kind ever experienced) and is labeled nonlinearly for intensity [26]. Subjects’ tolerance of the honey-based OVB taste (median 26.5 (IQR 15.0–47.5)) was greater than that of sucralose or xanthan. Sucralose and xanthan, however, were not significantly different in their scoring for taste (median scores: −5.0 (−17.5 to 18.5) vs. (median 7.5; IQR −10 to 20), respectively. It is difficult to compare the results of our study with the results obtained by Hefner et al. due to the use of different scales. Nevertheless, we can say that our formulation was rated no worse than honey (6.5/10 vs. 26/100, respectively).

Abnormal, only transient, cortisol levels were observed in only one patient. This is in line with the results of previous studies [27,28] and the results of a systematic review [29] that found abnormal cortisol values in a minority (16%) of patients taking topical corticosteroids for EoE. However, the authors of the systematic review emphasize that these are the results of uncontrolled, observational, heterogeneous studies, whereas in the randomized controlled trials, adrenal insufficiency did not occur more frequently with topical corticosteroids than with a placebo, albeit following a treatment interval of usually 12 weeks or less, nor was a decrease in serum cortisol noted in any of the observational cohorts that measured pre-treatment values.

Much research is still ongoing to develop an ideal budesonide carrier for the topical treatment of EoE. Most studies are based on the search for an ideal, viscous solvent ensuring the longest possible contact of the steroid with the esophageal mucosa. At the same time, new steroid-eluting esophageal-targeted drug delivery devices are also being tested, such as the fluticasone-eluting strings or fluticasone-loaded rings described by Prasher.

Our pilot study covered a comparatively small group of participants; however, the formulation was assessed not only based on its effectiveness but also on the opinions of patients regarding its taste and tolerance. The effectiveness of the slurry was measured in accordance with international standards described in the guidelines. The open-label character of the study as well as the lack of control group comparison makes our conclusions precarious. It means the necessity to confirm the results in further studies. The study was prospective, and the formulation studied is composed of hypoallergenic ingredients and is widely available, safe, and inexpensive.

## 6. Conclusions

Our proprietary formulation of budesonide solvent seems to have similar effectiveness to most popular sucralose-based viscous slurries used in trials to date. However, the small size of the study group and the heterogeneity of the population require a cautious approach to this statement. Due to the limitations of the study, such as the lack of a control group and the mentioned small size of the study group, we plan to confirm it in further research. The smell and consistency of the solvent were judged to be very good, and the overall tolerance to be good enough. This may suggest good compliance, which is of particular importance for the long-term treatment that EoE requires.

## Figures and Tables

**Table 1:** Taste evaluation results.

	N	SEX [M = 1]	AGE [Median, yrs]	Taste [Median]	Consistency [Median]	Smell [Median]	Ease of Swallowing [Median]	General Acceptance [Median]	8 Weeks QD [Y = 1]	12 Weeks QD [Y = 1]	8 Weeks BID [Y = 1]	12 Weeks BID [Y = 1]
**Doctors**	11	3	46	5	9	8	10	8	90% 9/10	89% 8/9	91% 10/11	87% 7/8
**Residents**	8	0	31	3.5	5.5	6.5	8	7	100% 8/8	100% 8/8	87% 7/8	87% 7/8
**Students**	16	2	23	4	8	8	8.5	6	94% 15/16	94% 15/16	94% 15/16	94% 15/16
**Nurses**	11	0	43	7	8	10	9	9	91% 10/11	91% 10/11	73% 8/11	73% 8/11
**Patients ≥ 150 cm**	7	6	13	3	6	10	9	6	57% 4/7	43% 3/7	57% 4/7	43% 3/7
**Patients < 150 cm**	3	3	10	4	6	8	10	8	100% 3/3	100% 3/3	100% 3/3	100% 3/3
**ADULTS**	46	5	28.5	4	8	8	8.5	7.5	93% 42/45	93% 41/44	87% 40/46	86% 37/43
**PATIENTS**	10	9	12.5	4	6	9	9	6.5	70% 7/10	60% 6/10	70% 7/10	60% 6/10

**Table 2:** Patients’ characteristics and results.

No. of Patient	AGE (y/o)	Height (cm)	before Intervention		after Intervention	
			Peak eos/HPF	ERFS (pts/10)	Peak eos/HPF	ERFS (pts/10)
**1.**	17	>150	30	3	0	1
**2.**	11	>150	20	5	2	1
**3.**	11	<150	20	2	50	6
**4.**	11	<150	40	2	0	0
**5.**	11	<150	20	0	20	2
**6.**	16	>150	80	3	0	2
**7.**	12	>150	40	6	0	1
**8.**	5	<150	30	5	0	0
**9.**	10	<150	40	3	0	2
**10.**	11	<150	30	1	60	2
**11.**	7	<150	30	4	20	1

## Data Availability

All data will be available from a corresponding author on a request.

## Supplementary Materials

The following supporting information can be downloaded at: https://www.mdpi.com/article/10.3390/jcm11226730/s1, Table S1: Study review. References [5,7,12,13,17,18,19,30,31,32,33,34,35] are cited in Supplementary Materials.
