# Supplementary material for: A New Viscous Budesonide Formulation for the Treatment of Eosinophilic Esophagitis in Children: A Preliminary Experience and Review of the Literature
Source: J Clin Med. 2022 Nov 14;11(22):6730. doi: 10.3390/jcm11226730 (PMC9694526; doi:10.3390/jcm11226730)
Supplement: Supplementary file 1 [file jcm-11-06730-s001.zip › jcm-2000683-supplementary.pdf]

Table S1. Study review

No.	Substance	Substance concentration	No. of patients treated with OVB	Age (y/o)	Sex (n, % male)	Treatment time	Total budesonide daily dosage	Responders (<15 eos/hpf)	Remission (<6 eos/hpf)	Pre-treatment peak eos/hpf (mean)	Post-treatment peak eos/hpf (mean)	Pre-treatment clinical symptomes	Post-treatment clinical symptomes	Pre-treatment endoscopic features	Post-treatment endoscopic features	Taste assesment
1	Sucralose	5g/dosage	30	1-17 (median: 8)	49 (80%)	6 weeks	1 mg (N= 15) 2 mg (N=15)	27 (90%)	N/A	56.5 [range 18-120]	3.9 [range 0-70]	N/A	N/A	N/A	N/A	N/A
	Applesauce	1 tbsp/dosage	19				0,5 mg (N = 1) 1 mg (N= 7) 2 mg (N=11)	15 (79%)		56.3 [range 20-100]	9.8 [range 0-40]					
	Honey	1 tbsp/dosage	7				1 mg (N= 2) 2 mg (N=5)	6 (86%)		53.7 [range 15-100]	8.6 [range 0-50]					
	Compound	N/A ("slurry" consistency)	1				1 mg	1		30	0					
	Hot cocoa		1				1 mg	1		30	0					
	Pear sauce		1				1 mg	1		30	0					
	Rice cereal		1				2 mg	0		100	41					
	Xanthan gum		1				2 mg	1		70	0					
2	Sucralose	5g/dosage	33	1-20 (mean: 10)	37 (77%)	8-12 weeks	1 mg (1-10 years) 2 mg (11-20 years)	Total 36 (75%)	Total 26 (54%) *<5 eos/hpf	45 ± 23	12 ± 16	N/A	N/A	44 (92%) (any abnormalities)	22 (46%) (any abnormalities)	N/A
	Neocate Duocal®	1 tbsp/dosage	11					sucralose (76%) Neocate Duocal (73%) Other(50%)	sucralose (58%) Neocate Duocal (55%) Other(50%)							
	Other (truvia, stevia, honey, maple syrup)	2 packets or 1 tablespoon	4													
3	Sucralose	10 g/1 mg budesonide	46	3.9-12.8 (median: 7.2)	37 (86.1%)	10 weeks-20 months	1mg (< 10 years) 2mg (> 10 years)	30 (65%)	23 (50%)	59.5 [range 20-180]	25.5 [range 0-200]	N/A	N/A	N/A	N/A	N/A
	Neocate Nutra®	2.5cm^2/1 mg budesonide	14	5.1-13.6 (median: 8.08)	13 (92.9%)	10 weeks - 6 months		13 (92.9%)	9 (64%)	62 [range 20-120]	9 [range 0-100]					
4	Sucralose	10g/1 mg budesonide	65	16-80 (mean: 36.2)	40 (62%)	8 weeks	2mg	40/56 (71%)	34/56 (61%) *<5 eos/hpf	72.6 ± 45.6	14.7 ± 29.0	DSQ score (mean) 10.6 ± 9.3	DSQ score (mean) 4.8 ± 7.3	EREFS (mean) 4.7	EREFS (mean) 2.1	N/A
5	Xylitol (mainly)	N/A	36	12.3 ± 7.3	21 (58.3%)	12 weeks	2mg (< 150cm) 4mg (>150cm)	partial responders *<19 eos/hpf 34 (94.4%)	32 (88.9%)	42.2 [range 15-100]	2.9 [range 0-30]	Author's Clinical Symptom Score (max. 14 pts.) 3.25 [range 0-9]	Author's Clinical Symptom Score (max. 14 pts.) 1.53 [range 0-7]	Endoscopy score (max. 15 pts.) 4.9 [range 0-9]	Endoscopy score (max. 15 pts.) 1.22 [range 0-6]	N/A
6	Sucralose	10g/1mg budesonide	15	1-17 (mean: 7.8)	12 (80%)	3 months	1 mg (< 5 ft.) 2mg (≥ 5 ft.)	partial responders *<19 eos/hpf 14 (93.3%)	13 (87%)	66.7 [range 22-125]	4.8 [range 0-25]	Symptom Scoring Tool (max. 14 pts.) 3.5 [range 0-10]	Symptom Scoring Tool (max. 14 pts.) 1.2 [range 0-7]	Endoscopy score (max. 15 pts.) 4.6 [range 0-9]	Endoscopy score (max. 15 pts.) 1.5 [range 0-7]	N/A
7	Sucralose	10 g/1 mg budesonide (approx. 10ml)	24	21-59 (median: 35.5)	13(54%)	single intervention	N/A	N/A	N/A	N/A	N/A	N/A	N/A	N/A	N/A	h-gLMS (-100 to 100 pts) 7.5
	Xanthan gum	50mg/1mg budesonide (approx. 10ml)	12	21-52 (median: 32.5)	6 (50%)											h-gLMS (-100 to 100 pts) 26.5
	Honey	5ml/1mg budesonide (approx. 10ml)	12	21-59 (median: 36)	7 (58%)											h-gLMS (-100 to 100 pts) -5.0
8	Sucralose	5g/1 mg budsonide	11	35 y/o (mean)	60% (of all participants)	8 weeks	2 mg	8 (73%)	8 (73%) (<7 eos/hpf)	83	11	MDQ-30 25 ±18	MDQ-30 16 ± 17	N/A	N/A	N/A
9	proprietary solution	N/A	17	1-18 (mean: 8.6)	14 (82.4%)	12 weeks	0.35 - 0.5 mg	N/A	4 (23.5%)	95.6	N/A	EoE CSS [max. 18] 7.4 (mean)	EoE CSS = 0 3/17 (17.6%)	N/A	N/A	N/A
			19	3-17 (mean: 10.2)	17 (89.5%)		1.4 - 2.0 mg		10 (52.6%)	107.7		EoE CSS [max. 18] 6.1 (mean)	EoE CSS = 0 6/19 (31.6%)			
			17	2-17 (mean: 7.9)	13 (76.5%)		2.8 - 4.0 mg		16 (94.1%)	108.4		EoE CSS [max. 18] 7.2 (mean)	EoE CSS = 0 3/17 (17.6%)			
10	Proprietary solution	N/A	19	mean: 46.5	14 (73.7%)	2 weeks	4 mg	18 (94.7%) (<16 eos/hpf)	N/A	201	21	N/A	N/A	modified EREFS 6.9	modified EREFS 3.2	N/A
11	Sucralose or honey	N/A	22 adults	2- 64 (median: 33)	84%	8 weeks	2 mg	15 (56%)	N/A	N/A	N/A	N/A	N/A	N/A	N/A	N/A
			5 children				1 mg									
12	Sucralose	"aqueous gel" consistency	25	mean: 39	19 (76%)	6 weeks	2 mg	N/A	23 (92%)	28-31	0	N/A	N/A	N/A	N/A	N/A
13	Proprietary solution (mainly dextrose)	N/A	213	11-55 (mean: 33.8)	129 (60.6%)	12 weeks	4 mg	N/A	113 (53.1%)	no. of participants with <15 eos/hpf after treatment 123 (62%)		DSQ score reduction ≥ 30% 112/213 (52.6%)		least-squares mean change in total EREFS - 4.0		N/A
14	Proprietary MB-9 formulation	N/A	33 adults	11-40 (mean: 22.3)	35 (68.6%)	12 weeks	4 mg	N/A	19 (39%)	156.3	39.3	DSQ score 29.3	DSQ score 15.0	EREFS 7.7 (mean)	EREFS 3.9 (mean)	N/A
			18 children													

OVB - oral viscous budesonide, eos/hpf - eosinophils per high power field, tbsp - tablespoon, DSQ - Dysphagia Symptom Questionnaire, EREFS - Endoscopic Reference Score, h-gLMS - hedonics generalized Labeled Magnitude Scale, EoE CSS - Eosinophilic Esophagitis Clinical Symptome Score;

1. J, Shuker M, Brown-Whitehorn T, Cianferoni A, Gober L, Muir A, Verma R, Liacouras C, Spergel JM. **Oral viscous budesonide can be successfully delivered through a variety of vehicles to treat eosinophilic esophagitis in children.** *J Allergy Clin Immunol Pract.* 2016 Jul-Aug;4(4):767-8. doi: 10.1016/j.jaip.2016.02.005. Epub 2016 Mar 26. PMID: 27025298.

2. Fable, J. M.; Fernandez, M; Goodine, S; Lerer, T; Sayej, W. N.,. **Retrospective Comparison of Fluticasone Propionate and Oral Viscous Budesonide in Children With Eosinophilic Esophagitis.** *Journal of Pediatric Gastroenterology and Nutrition: January 2018 - Volume 66 - Issue 1 - p 26-32 doi: 10.1097/MPG.0000000000001626*

3. *Rubinstein, E.; Lee, J. J.; Fried, A.; Logvinenko, T.; Ngo, P.; McDonald, D.; Hait, E. J.* ***Comparison of 2 Delivery Vehicles for Viscous Budesonide to Treat Eosinophilic Esophagitis in Children.*** *Journal of Pediatric Gastroenterology and Nutrition* : September 2014 - Volume 59 - Issue 3 - p 317-320
doi: 10.1097/MPG.0000000000000436

4. Dellon ES, Woosley JT, Arrington A, McGee SJ, Covington J, Moist SE, Gebhart JH, Tylicki AE, Shoyoye SO, Martin CF, Galanko JA, Baron JA, Shaheen NJ. **Efficacy of Budesonide vs Fluticasone for Initial Treatment of Eosinophilic Esophagitis in a Randomized Controlled Trial.** *Gastroenterology.* 2019 Jul;157(1):65-73.e5. doi: 10.1053/j.gastro.2019.03.014. Epub 2019 Mar 11. PMID: 30872104; PMCID: PMC6581596.

5. Oliva S, Rossetti D, Papoff P, Tiberti A, Rossi P, Isoldi S, Amil Dias J, Lucarelli S, Cucchiara S. **A New Formulation of Oral Viscous Budesonide in Treating Paediatric Eosinophilic Oesophagitis: A Pilot Study.** *J Pediatr Gastroenterol Nutr.* 2017 Feb;64(2):218-224. doi: 10.1097/MPG.0000000000001281. PMID: 27253660.

6. Dohil R, Newbury R, Fox L, Bastian J, Aceves S. **Oral viscous budesonide is effective in children with eosinophilic esophagitis in a randomized, placebo-controlled trial.** *Gastroenterology* . 2010 Aug;139(2):418-29. doi: 10.1053/j.gastro.2010.05.001. Epub 2010 May 7. PMID: 20457157.

7. Hefner, J.N., Howard, R.S., Massey, R. et al. **A Randomized Controlled Comparison of Esophageal Clearance Times of Oral Budesonide Preparations.** *Dig Dis Sci* 61, 1582–1590 (2016). https://doi.org/10.1007/s10620-015-3990-4

8. Dellon ES, Sheikh A, Speck O, Woodward K, Whitlow AB, Hores JM, Ivanovic M, Chau A, Woosley JT, Madanick RD, Orlando RC, Shaheen NJ. **Viscous topical is more effective than nebulized steroid therapy for patients with eosinophilic esophagitis.** *Gastroenterology* . 2012 Aug;143(2):321-4.e1. doi: 10.1053/j.gastro.2012.04.049. Epub 2012 May 3. PMID: 22561055; PMCID: PMC3404241.

9. Gupta SK, Vitanza JM, Collins MH. **Efficacy and safety of oral budesonide suspension in pediatric patients with eosinophilic esophagitis.** *Clin Gastroenterol Hepatol.* 2015 Jan;13(1):66-76.e3. doi: 10.1016/j.cgh.2014.05.021. Epub 2014 Jun 4. PMID: 24907502.

10. Miehlke S, Hruz P, Vieth M, Bussmann C, von Arnim U, Bajbouj M, Schlag C, Madisch A, Fibbe C, Wittenburg H, Allescher HD, Reinshagen M, Schubert S, Tack J, Müller M, Krummenerl P, Arts J, Mueller R, Dilger K, Greinwald R, Straumann A. **A randomised, double-blind trial comparing budesonide formulations and dosages for short-term treatment of eosinophilic oesophagitis.** *Gut* . 2016 Mar;65(3):390-9. doi: 10.1136/gutjnl-2014-308815. Epub 2015 Mar 19. PMID: 25792708; PMCID: PMC4789829.

11. Albert D, Heifert TA, Min SB, Maydonovitch CL, Baker TP, Chen YJ, Moawad FJ. **Comparisons of Fluticasone to Budesonide in the Treatment of Eosinophilic Esophagitis.** *Dig Dis Sci.* 2016 Jul;61(7):1996-2001. doi: 10.1007/s10620-016-4110-9. Epub 2016 Apr 19. PMID: 27093866.

12. Philpott, H., Nandurkar, S., Royce, S.G., Thien, F. and Gibson, P.R. (2016), **A prospective open clinical trial of a proton pump inhibitor, elimination diet and/or budesonide for eosinophilic oesophagitis.** *Aliment Pharmacol Ther* , 43: 985-993. https://doi.org/10.1111/apt.13576

13. Hirano I, Collins MH, Katzka DA, Mukkada VA, Falk GW, Morey R, Desai NK, Lan L, Williams J, Dellon ES; ORBIT1/SHP621-301 Investigators. **Budesonide Oral Suspension Improves Outcomes in Patients With Eosinophilic Esophagitis: Results from a Phase 3 Trial.** *Clin Gastroenterol Hepatol.* 2022 Mar;20(3):525-534.e10. doi: 10.1016/j.cgh.2021.04.022. Epub 2021 Apr 19. Erratum in: Clin Gastroenterol Hepatol. 2022 Oct;20(10):2418. PMID:33887475.

14. Dellon ES, Katzka DA, Collins MH, Hamdani M, Gupta SK, Hirano I; MP-101-06 Investigators. **Budesonide Oral Suspension Improves Symptomatic, Endoscopic, and Histologic Parameters Compared With Placebo in Patients With Eosinophilic Esophagitis.** *Gastroenterology* . 2017 Mar;152(4):776-786.e5. doi: 10.1053/j.gastro.2016.11.021. Epub 2016 Nov 23. PMID: 27889574.
